# Nrf2 participates in mechanisms for reducing the toxicity and enhancing the antitumour effect of Radix *Tripterygium wilfordii* to S180-bearing mice by herbal-processing technology

**DOI:** 10.1080/13880209.2019.1634106

**Published:** 2019-07-07

**Authors:** Jun-Ming Wang, Jin-Yang Li, Hong Cai, Rong-Xing Chen, Yue-Yue Zhang, Lu-Lu Zhang, Ying Cui, Yong-Xian Cheng

**Affiliations:** a College of Pharmacy, Henan University of Chinese Medicine, Zhengzhou, China;; b Collaborative Innovation Center for Respiratory Disease Diagnosis and Treatment & Chinese Medicine Development of Henan Province, Henan University of Chinese Medicine, Zhengzhou, China;; c Health Science Center, Shenzhen University, Shenzhen, China

**Keywords:** *Lonicera japonica* Thunb, *Lysimachia christinae* Hance, *Glycyrrhiza uralensis* Fisch, *Paeonia lactiflora* Pall, *Phaseolus radiatus* L

## Abstract

**Context:** Radix *Tripterygium wilfordii* Hook. f. (Celastraceae) (LGT) has outstanding curative efficacy; however, side effects include high toxicity, particularly hepatotoxicity and nephrotoxicity.

**Objective:** To investigate detoxification mechanisms of LGT through processing separately with each of these medicinal herbs including Flower *Lonicera japonica* Thunb. (Caprifoliaceae) (JYH), Radix *Paeonia lactiflora* Pall. (Ranunculaceae) (BS), Herba *Lysimachia christinae* Hance (Primulaceae) (JQC), Radix et Rhizoma *Glycyrrhiza uralensis* Fisch. (Fabaceae) (GC) and Seed *Phaseolus radiatus* L. (Fabaceae) (LD) in S180-bearing mice by involving nuclear factor (erythroid-derived 2)-like 2 (Nrf2).

**Materials and methods:** LGT raw and processed products were orally administered at 60 mg/kg to KM male mice inoculated with S180 tumour cells for 14 consecutive days, and blood, tumour, liver and kidney were taken to observe the detoxifying effects and biological mechanisms.

**Results:** Herbal-processing technology significantly weakened hepatotoxicity and nephrotoxicity evoked by LGT with ED_50_ of the converted triptolide in each processed-herb product for serum alanine transaminase, aspartate transaminase, creatinine and urea nitrogen of 9.3, 16.6, 2.5 and 4.2 μg/kg, for liver glutathione, glutathione *S*-transferase, catalase, tumour necrosis factor-α and interleukin-10 of 114.9, 67.8, 134.1, 7.7, 4171.6 μg/kg, and for kidney 21.9, 20.5, 145.0, 529.7, 19.4 μg/kg, respectively. Moreover, herbal-processing technology promoted the accumulation of Nrf2 into the nucleus, and upregulated mRNA expression of *Nrf2* and *heme oxygenase-1*. Additionally, herbal-processing technology enhanced the tumour inhibition rate with ED_50_ 12.2 μg/kg.

**Discussion and conclusions:** Herbal-processing technology improves the safety and effectiveness of LGT in cancer treatment, and future research may be focused on the Nrf2-related molecules.

## Introduction

Processing (‘Paozhi’ in Chinese Pinyin), as an ancient and classic pharmaceutical technology of traditional Chinese medicine (TCM), is one of the characteristics and advantages of TCM (Wang et al. 2012a; Cai et al. 2017). According to the TCM theory, proper processing can reduce the toxicity and change the curative efficacy of Chinese herbal medicine through proper processing (CHM) (Wang et al. 2012a; Cai et al. 2017). Processed-herb technology, as one of the most common and traditional concocted techniques, is to use some CHMs to concoct other CHMs in order to promote efficacy, attenuate toxicity, prevent the poor bias or influence medicinal properties (Wang et al. 2012a; Cai et al. 2017). Since ‘Toxicity of Radix *Aconitum carmichaelii* Debx. (Ranunculaceae) (Chuanwu) is alleviated by processing with a medicinal herb *Apis cerana* Fabricius (Apidae) (Fengmi)’ (Cai et al. 2017) was first recorded by Zhang Zhongjing’s ‘Synopsis of Prescriptions of the Golden Chamber’ in the Eastern Han Dynasty of China, processed-herb technique has been used for more than 2000 years and has been continuously enriched and improved in modern applications such as *Zingiber officinale* Rosc. (Zingiberaceae) (Shengjiang)-processed *Pinellia ternata* (Thunb.) Breit. (Araceae) (Banxia) and cortex *Magnolia officinalis* Rehd. et Wils. (Magnoliaceae) (Houpo), Fengmi-processed Radix et Rhizoma *Glycyrrhiza uralensis* Fisch. (Fabaceae) (Gancao, GC) and *Lilium brownii* F.E. Brown var. *viridulum* Baker (Liliaceae) (Baihe), *Euodia rutaecarpa* (Juss.) Benth. (Rutaceae) (Wuzhuyu)-processed *Coptis chinensis* Franch. (Ranunculaceae) (Huanglian), Typhae pollen (Puhuang)-processed Asini Corii Colla (Ejiao) (Cai et al. 2017) and so on. However, the principle and essence on processed-herb technology are almost unknown, which restricts its reasonable application. In recent years, research (Wu et al. 2012; Chen et al. 2013; Gong et al. 2013; Zhao et al. 2014; Cao et al. 2015b; Yun et al. 2015) on the processed detoxification of toxic CHMs, such as Radix *Euphorbia kansui* T.N. Liou ex T.P. Wang (Euphorbiaceae) (Gansui), Euphorbiae Radix *Euphorbia pekinensis* Rupr. (Euphorbiaceae) (Jingdaji), Flos *Daphne genkwa* Sieb. et Zucc. (Thymelaeaceae) (Yuanhua), Radix *Phytolacca acinosa* Roxb. (Phytolaccaceae) (Shanglu), Radix Praeparaia *Aconitum carmichaelii* Debx. (Ranunculaceae) (Fuzi) and Radix *Polygonum multiflorum* Thunb. (Polygonaceae) (Heshouwu), has become one of the hot topics in academia.

Radix *Tripterygium wilfordii* Hook. f. (Celastraceae) (Leigongteng, LGT) is first recorded in one of the oldest books on the foundation of TCM, ‘Shen Nong's Herbal Classic’ (Liu et al. 2017), which was published over two thousand years ago. LGT possess common characteristics with a bitter/pungent flavour and a cold nature (Liu et al. 2017) and has been used as a traditional oriental CHM for centuries to treat a variety of cancers (Wang et al. 2016a; Liu et al. 2009), diabetic nephropathy (Ge et al. 2013), rheumatoid arthritis (Bao and Dai 2011) and so on. However, it can also often cause multiple organs toxicities (Wang et al. 2012b, 2012c; Cao et al. 2015a; Li et al. 2015; Liu et al. 2017), hepatotoxicity and nephrotoxicity (Li et al. 2015) in particular, due to an overdose or prolonged exposure in clinic. A significant increase in serum alanine transaminase (ALT) and aspartate transaminase (AST) is usually indicative of hepatotoxicity (Li et al. 2015), and increase in serum creatinine (Cr) and urea nitrogen (BUN) is usually indicative of nephrotoxicity (Li et al. 2015).

At present, research on the processed-herb detoxification of LGT is rare. Only a few studies (Ma et al. 2017; Zhao et al. 2017) mainly focused on the GC-processed detoxification actions of LGT *in vivo* and *in vitro*, but these studies did not elucidate the detoxification mechanisms. In this context, we tried to select Chinese medicinal herbs to concoct LGT under the guidance of TCM theory, thereby reducing the toxicity of LGT without reducing or even enhancing its curative efficacy. In TCM theory, sweetness in five flavours of Chinese medicine has the functions of relieving toxicity, relieving food poisoning and alleviating drastic drug properties. GC, Flower *Lonicera japonica* Thunb. (Caprifoliaceae) (Jinyinhua, JYH), Seed *Phaseolus radiatus* L. (Fabaceae) (Lvdou, LD), Herba *Lysimachia christinae* Hance (Primulaceae) (Jinqiancao, JQC) and Radix *Paeonia lactiflora* Pall. (Baishao, BS) have sweet properties in five flavours of Chinese medicine. Among them, GC, LD and JYH are commonly used to alleviate or prevent poisoning from drugs or foods (Song 1991; Yang 2016; Fei et al. 2017), JQC can be often administered to alleviate poisoning from LGT (Wang et al. 2016b; Liu et al. 2017), and BS is commonly used to antagonize LGT-induced toxicity (Li et al. 2009). In addition, studies have shown that JYH, BS, JQC, GC and LD and the main active extracts and compounds they contain have hepatoprotective and (or) kidney-protective effects (Sohn et al. 2003; Sun et al. 2008, 2010; Wang et al. 2012d; Liu et al. 2015; Yagmurca et al. 2015; Jung et al. 2016; Xin et al. 2016; Wang et al. 2016c; Ye et al. 2017; Xie et al. 2018). Therefore, considering it comprehensively, we chose above five medicinal herbs including JYH, BS, JQC, GC and LD for processing LGT to evaluate its detoxification effects. Our previous study (Wang et al. 2018a) has evaluated the chemical basis of the detoxification effects of processed-herb LGT and confirmed that the processing could significantly reduce the contents of TP and CEL of the main toxic components contained in LGT, and the chemical total score of 11 different characteristic components including TP and CEL was also significantly reduced. As mentioned earlier, the toxic target organs of LGT are mainly in the liver and kidneys (Li et al. 2015). Therefore, our other study also observed and confirmed the detoxification effects of LGT by processing with medicinal herbs under physiological conditions (Wang et al. 2017). Further, considering that LGT can be used in many kinds of cancers (Liu et al. 2009; Cao et al. 2015a; Wang et al. 2016a), and that in the clinic, the drug is administrated to patients instead of healthy people, our intent was to observe the detoxification effects of LGT via processing with medicinal herbs including JYH, GC, JQC, LD and BS under the pathological state of tumour.

In addition, it has been reported that LGT-induced toxicity is related to oxidative stress and inflammation damage (Li et al. 2015; Wang et al. 2015), while JYH, BS, JQC, GC and LD all have been reported to be of antioxidant and anti-inflammatory properties (Gu et al. 1988; Lee et al. 2005; He and Dai 2011; Wu et al. 2011; Chen et al. 2012; Guo et al. 2014; Luo et al. 2016; Tóth et al. 2016). Therefore, we speculated that these five herbs may probably detoxify LGT through antioxidant and anti-inflammatory processes. Moreover, considering nuclear factor (erythroid-derived 2)-like 2 (Nrf2), an antioxidant key transcription factor, plays an important role in both antioxidant and anti-inflammatory processes (Wang et al. 2018b), we investigated the detoxification mechanisms of LGT via processing with medicinal herbs based on Nrf2 antioxidant and anti-inflammatory defences.

## Materials and methods

### Experimental animals

Considering that there is a gender difference in the toxicity of LGT, and that oestrogens in female animals may probably interfere with the effects of drugs (Wang et al. 2018b), male mice are used as research animals in this study. Kunming (KM) male mice (18–22 g) were obtained from Experimental Animal Center of Henan Province (Zhengzhou, China). Animals were given rodent laboratory chow and water *ad libitum* and maintained under controlled conditions with a temperature of 22 ± 1 °C, relative humidity of 60% ± 10% and a 12-h light/dark cycle (lights on at 7:00 AM). All the procedures were in strict accordance with the P.R. China legislation on the use and care of laboratory animals and guidelines formulated by the Institute for Experimental Animals of Henan University of Chinese Medicine and were approved by the university committee for animal experiments.

### Cell lines

Mouse S180 tumour cells were collected from S180 tumour-bearing mice which were purchased from the Institute of Chinese Materia Medica, China Academy of Chinese Medical Sciences (Beijing, China). Mouse S180 tumour cells were maintained in the peritoneal cavities of male KM mice in the Laboratory of Experimental Animals of Henan University of Chinese Medicine (Zhengzhou, China).

### Reagents

Kits including alanine/aspartate transaminase (ALT/AST), creatinine (Cr), urea nitrogen (BUN), glutathione (GSH), glutathione s-transferase (GST), glutathione peroxidase (GPx), superoxide dismutase (SOD), catalase (CAT) and Bradford protein assay were all purchased from Nanjing Jiancheng Bioengineering Institute (Nanjing, China). Mouse tumour necrosis factor-alpha (TNF-α) and interleukin (IL)-10 enzyme-linked immunosorbent assay (ELISA) kits were provided by Boster Biological Technology (Wuhan, China). Besides, triptolide (TP) and celastrol (CEL) were purchased from Chengdu Manchester Biotechnology Co., Ltd. (Chengdu, China). The batch numbers of TP and CEL were MUST-15081405 and MUST-15082610, respectively. The high-performance liquid chromatography (HPLC) purities were 99.78% and 99.79%, respectively.

### Plant material and preparations of LGT-processed products

LGT was obtained from Taining County of Fujian Province (Taining, China). JYH, BS, JQC and GC were all purchased from a traditional drug store, Henan Materia Medica Chain Co., Ltd. (Zhengzhou, Henan province, P.R. China). LD was purchased from Zhengzhou Wal-Mart Supermarket (Manhattan Store). LGT, LJYH, BS, JQC, GC and LD were all identified by Prof. Sui-Qing Chen (Pharmacognosy Department, Henan University of Chinese Medicine, Zhengzhou, Henan province, P.R. China).

The method of concocting LGT is detailed in our recent study (Wang et al. 2018a), which is briefly described below. Separately weigh the appropriate amounts (such as 10 g) of five kinds of processed auxiliary materials (JYH, BS, JQC, GC and LD), place them in different stainless steel pots, add 20 times the amount of water (w/v = 1:20) for 0.5 h, then boil 2 times, each time 15–20 min. Subsequently, the decoctions were combined and concentrated to processed auxiliary liquids each with a concentration of 0.05 g/mL. Weigh several portions of LGT raw materials (such as 60 g) separately, add 0.05 g/mL each of the processed auxiliary liquids (such as 200 mL), place them in a stainless steel pot, cook them with slow fire until the medicine is thoroughly saturated and drained, remove them and dry in the far-infrared constant temperature oven (60 °C). Finally, we obtained LGT-processed products, namely, JYH-processed LGT (JYHLGT), BS-processed LGT (BSLGT), JQC-processed LGT (JQCLGT), GC-processed LGT (GCLGT), LD-processed LGT (LDLGT). Note: For each 60 g LGT decoction pieces, use auxiliary materials JYH, BS, JQC, GC, LD each 10 g.

We further determined the difference in chemical composition of LGT before and after processing by HPLC and combined the methods of principal component analysis and grey correlation analysis to evaluate the chemical basis of the detoxification effects of LGT (Wang et al. 2018a). The contents of TP and CEL in LGT and its processed products JYHLGT, BSLGT, JQCLGT, GCLGT and LDLGT were determined as 0.345, 0.038, 0.062, 0.030, 0.118, 0.052 mg/g (for TP), and 6.399, 0.973, 0.652, 0.235, 0.362, 0.834 mg/g (for CEL), respectively, by the HPLC analysis (Wang et al. 2018a). In addition, we also found 11 different characteristic components (including TP and CEL) that changed before and after processing, and reduced the dimensions of the 11 features by principal component analysis and calculated it as a single chemical score that can basically represent the composition of the components. The chemical scores of the processed products were reduced by 170.2, 88.6, 128.0, 59.4 and 153.8%, respectively, compared with the LGT raw product (Wang et al. 2018a).

### Animal experiment-related treatment protocol

Considering that ethyl acetate can dissolve LGT-contained major bioactive compounds such as TP and CEL, and ethyl acetate is often used for an extraction solvent to extract LGT in a variety of researches (Tao et al. 2001; Bai et al. 2003; Tao et al. 2006), so we selected ethyl acetate for extraction solvent to prepare all the extracts through conventional reflux extraction. The preparations of the ethyl acetate extracts from LGT raw product and its processed-herb products with JYH, BS, JQC, GC and LD were described as follows. Ethyl acetate extracts (EAEs) from LGT and the processed-herb products including JYHLGT, BSLGT, JQCLGT, GCLGT and LDLGT were obtained by reflux extraction once 2 h for repeated three times. The ethyl acetate was recovered under reduced pressure to a thick paste, and the thick paste was placed on a water bath at 85 °C (a temperature slightly above the boiling point of ethyl acetate) to heat off the odour of ethyl acetate and then dried under vacuum at 60 °C to obtain a dry extract without ethyl acetate. The contents of TP and CEL in extracts from LGT and its processed products JYHLGT, BSLGT, JQCLGT, GCLGT and LDLGT are determined as 1.306, 0.609, 0.581, 0.366, 1.029, 0.454 mg/g (for TP), and 27.060, 7.804, 5.378, 2.084, 3.574, 5.766 mg/g (for CEL), respectively, by the HPLC analysis.

According to the literature method, S180 ascites tumour cells were inoculated into the right armpit of mice to prepare S180 solid tumour model (Wang et al. 2018b). One day after inoculation, mice, except for the normal (non-tumour-inoculated) animals, were randomly divided into eight groups of 10 mice each. The control (tumour-inoculated) groups of mice received daily oral administration of 0.5% (5 g/L) sodium carboxyl methyl cellulose as a suspending agent (Meler and Wendt 1990) (CMC-Na; 0.2 mL/10 g). The other six groups received LGT raw product and processed-herb products (JYHLGT, BSLGT, JQCLGT, GCLGT and LDLGT) by intragastric administration (*ig*) for 14 d starting from 24 h after tumour inoculation. Our previous study (Wang et al. 2018b) had confirmed that LGT raw product could cause toxicity in S180 tumour-bearing mice when administered at a dose of 60 mg/kg (equivalent to LGT crude drug about 2 g/kg). Therefore, all doses of LGT for raw and processed products in this study were set at 60 mg/kg, ensuring that all doses were comparable at the same level. After treatment, mice were sacrificed by cervical dislocation after peripheral blood samples, livers, kidneys and tumours were collected at 24 h after the last administration. Serum samples were collected for the analysis of biochemical indicators including ALT, AST, Cr and BUN, and liver and kidney tissues were used for the analysis of the histological observation, GSH, TNF-α and IL-10 levels, determination of glutathione-related and antioxidant enzymes. The tumours were weighed, arrayed in line on paper, and taken pictures. The tumour inhibition rate was calculated by the formula of IR = [(C − T)/C] × 100%, where C and T are the mean tumour weights of the control group (CMC-Na) and the treated group, respectively.

In addition, it was worth noting that in order to overall reflect the attenuation and synergistic effect of the herbal-processing technology on LGT through the effective half dose (ED_50_) value, we calculated the dose of TP according to the TP content in each processed product and obtained the ED_50_ value according to the biological activity and the converted TP dose of each processed product.

### Assay for serum ALT, AST, Cr and BUN

Blood samples were obtained from mice of all groups (ten mice per group) for the determination of serum biochemical biomarkers. Serum ALT, AST, Cr and BUN were assayed by the commercial kits (Nanjing Jiancheng Bioengineering Institute, Nanjing, China) according to the manufacturer’s protocols.

### Histological observation

After fixation in 10% formalin, the livers and kidneys were examined for size, colour changes and haemorrhage. Slices of liver and kidney were cut into small pieces and histological sections were stained with haematoxylin and eosin (H&E) for the observation under the 200 × light microscopy.

### Assay for levels of protein, antioxidants, IL-10 and TNF-α

Liver and kidney tissues were homogenized in cold physiological saline, and their total protein concentrations were measured by the commercial Bradford Assay Kit (Nanjing Jiancheng Bioengineering Institute, Nanjing, China) according to the manufacturer’s protocol. Hepatic and kidney tissues levels of antioxidants including GSH, GST, GPx, SOD and CAT and inflammatory mediators including IL-10 and TNF-α were assayed, respectively, by the commercial kits (for antioxidants Nanjing Jiancheng Bioengineering Institute, Nanjing, China) and (for inflammatory mediators Boster Biological Technology, Wuhan, China) according to the manufacturer’s protocols, and the results were all expressed based on tissue protein concentrations which were measured by Bradford Protein Assay.

### Western blot analysis

Liver and kidney proteins were separated as described in the Nuclear Extraction Reagent Kit (Pierce, USA). The protein concentrations were measured, and all the samples in the same experiment were normalized to the equal protein concentration. Protein samples were isolated by SDS-PAGE gel electrophoresis and transferred onto a PVDF membrane, and then incubated with the appropriate combination of primary and secondary antibodies, followed by ECL detection and quantification using an image analysis program. The grey densities of the protein bands were normalized by using Lamin B density as internal control, and the results were further normalized to normal control.

### Real-time PCR analysis

Total RNA was extracted from liver and kidney tissues using TRIzol reagent following the manufacturer’s instructions. cDNA was synthesized and real-time PCR was conducted as described in kits. The PCR primer sequences were as follows: Nrf2 forward 5′-TTCCTCTGCTGCCATTAGTCAGTC-3′, reverse 5′-GCTCTTCCATTTCCGAGTCACTG-3′ (242 bp product with Accession No. NM_010902.4 synthesized from Mus musculus); heme oxygenase-1 (HO-1) forward 5′-ACGCATATACCCGCTACCTG-3′, reverse 5′-CCAGAGTGTTCATTCGAGCA-3′ (174 bp product with Accession No. NM_010442.2 synthesized from Mus musculus); and glyceraldehydes-3-phosphate dehydrogenase (GAPDH) forward 5′-CAAGGTCATCCATGACAACTTTG-3′, reverse 5′-GGGCCATCCACAGTCTTCTG-3′ (90 bp product with Accession No. NM_008084.3 synthesized from Mus musculus). Relative expression of target genes was normalized to GAPDH, analyzed by 2^-ΔΔCt^ method and given as ratio compared with the normal control.

### Statistical analysis

The results were presented as mean ± standard deviation of mean (SD). The differences among experimental groups were compared by one-way ANOVA (analysis of variance), followed by least significant difference (LSD) when the variance is equal or by Dunnett T3 when the variance is not uniform, using the SPSS (Statistics Package for Social Science) program version 17.0. *p* < 0.05 indicated statistical difference.

## Results

### Processing with herbs reversed LGT-induced hepatotoxicity and nephrotoxicity

**Figure 1.**
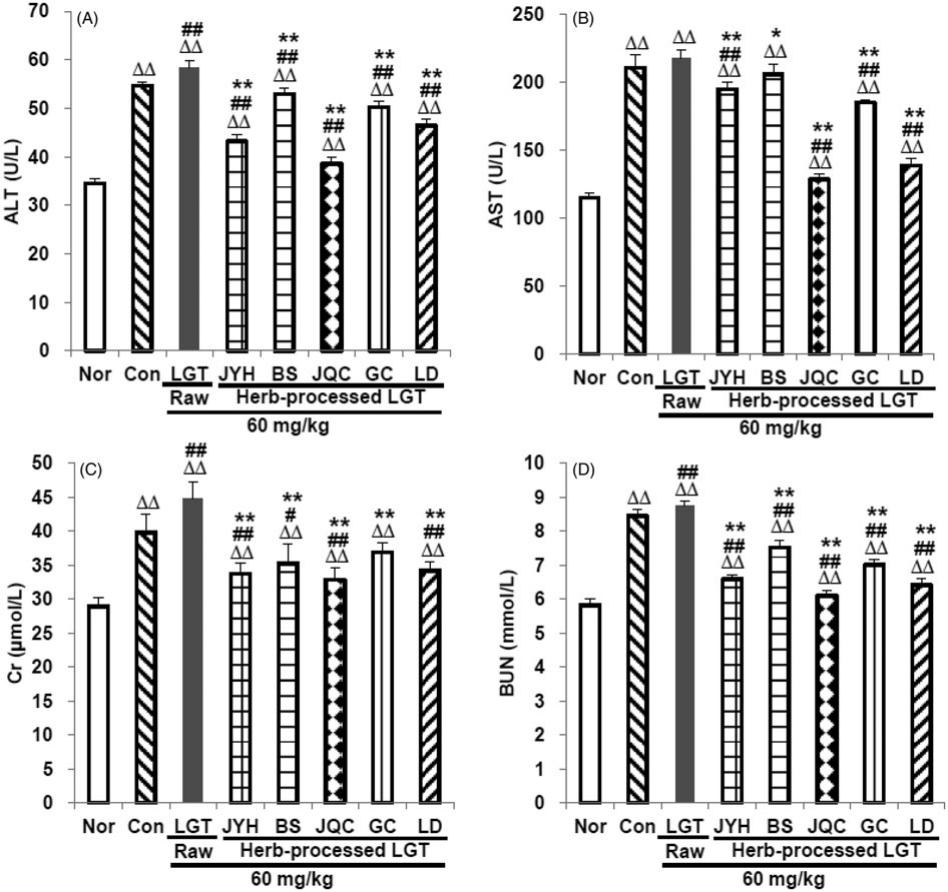
LGT processed with JYH, BS, JQC, GC and LD was used to treat LGT-exposed S180 tumour-bearing mice. The serum ALT (A), AST (B), Cr (C) and BUN (D) levels were subsequently examined. Data are presented as mean ± SD (*n* = 10). Significant differences compared with the normal (Nor) group were designated as ΔΔ*p* < 0.01, with the control (Con) group as #*p* < 0.05 and ##*p* < 0.01, and with LGT raw product group as *p < 0.05 and **p < 0.01.

**Figure 2.**
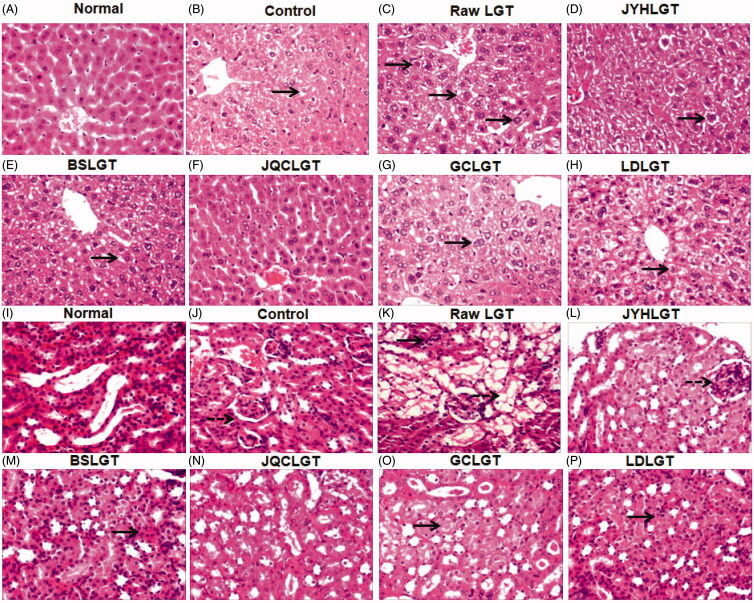
Effects of the processed LGT on liver (from A to H) and kidney (from I to P) pathology by H&E × 200 in LGT-exposed S180 tumour-bearing mice. Black solid arrows indicate swelling-like degeneration of hepatocytes or nephrocytes, and black dotted arrows indicate inflammation of nephrocytes.

In this study, compared with the normal (Nor) group, inoculation of S180 tumour in the control (Con) group significantly elevated serum ALT, AST, Cr and BUN levels (all *p* < 0.01) (Figure 1(A–D)), while administration of LGT raw product (unprocessed) further raised the levels of the above indicators in S180 tumour-bearing mice, among which three indicators including ALT (58.4 U/L), Cr (44.8 μmol/L) and BUN (8.7 mmol/L) are statistically different (all *p* < 0.01) (Figure 1(A,C,D)), suggesting that LGT raw product treatment for 14 d evoked subacute hepatotoxicity and nephrotoxicity in S180 tumour-bearing mice. In contrast, treatment with the processed-products of LGT (processing with JYH, BS, JQC, GC and LD, respectively) for 14 d effectively weakened LGT-induced hepatotoxicity and nephrotoxicity with each ED_50_ according to the converted TP in above processed products for serum ALT, AST, Cr and BUN of 9.3, 16.6, 2.5 and 4.2 μg/kg, respectively. Further, histological evaluation of the livers and kidneys removed from S180-bearing mice administrated with LGT raw product demonstrated swelling-like degeneration and (or) inflammation of hepatocytes and nephrocytes (Figure 2) as shown in black solid arrows indicating swelling-like degeneration of hepatocytes or nephrocytes, and black dotted arrows indicating inflammation of nephrocytes. After treatment with processed-herb products including JYHLGT, BSLGT, JQCLGT, GCLGT and LDLGT, these abnormal changes conspicuously decreased or even disappeared (Figure 2). These results suggested that processing with herbs including JYH, BS, JQC, GC and LD all reversed hepatotoxicity and nephrotoxicity induced by LGT in S180 tumour-bearing mice.

### Processing with herbs reversed LGT-decreased antioxidant levels in liver and kidney

**Figure 3.**
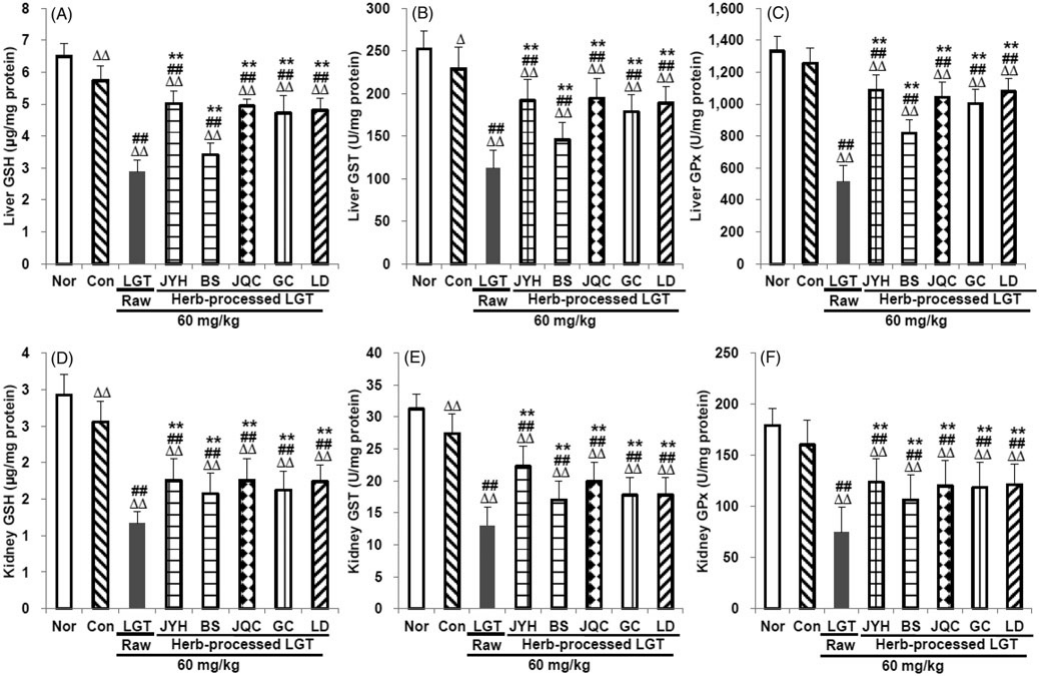
Effects of processed LGT treatment on glutathione-related antioxidants GSH (A,D), GST (B,E) and GPx (C,F) levels in liver and kidney of LGT-exposed S180 tumour-bearing mice. Significant differences compared with the normal (Nor) group were designated as Δ*p* < 0.05 and ΔΔ*p* < 0.01, with the control (Con) group as ##*p* < 0.01, and with LGT raw product group as **p < 0.01.

**Figure 4.**
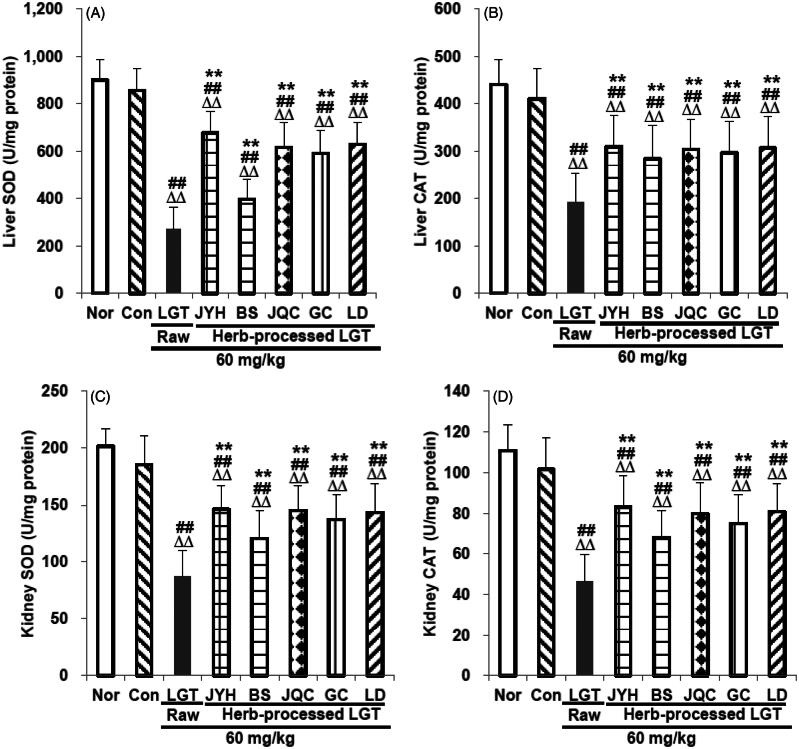
Effects of processed LGT treatment on primary antioxidant enzymes SOD (A,C) and CAT (B,D) levels in liver and kidney of LGT-exposed S180 tumour-bearing mice. Significant differences compared with the normal (Nor) group were designated as ΔΔ*p* < 0.01, with the control (Con) group as ##*p* < 0.01, and with LGT raw product group as **p < 0.01.

In this study, compared with the Nor group, inoculation of S180 tumour in the Con group significantly reduced GSH and GST levels in mice liver (*p* < 0.01 and *p* < 0.05, respectively) (Figure 3(A,B)) and kidney (both *p* < 0.01) (both *p* < 0.01) (Figure 3(D,E)) without obvious effects on GPx (Figure 3(C,F)), SOD (Figure 4(A,C)) and CAT (Figure 4(B,D)), either in the liver or in the kidney. Compared with the Con group, administration of LGT raw product further significantly reduced the levels of GSH, GST, GPx, SOD and CAT both in liver and kidney of the S180-bearing mice (all *p* < 0.01) (Figures 3(A–F) and 4(A–D)). Compared with the LGT raw product group, processing with herbs including JYH, BS, JQC, GC and LD all significantly reversed the excessively low levels of all above indicators (all *p* < 0.01) induced by LGT in liver and kidney (Figures 3 (A–F) and 4(A–D)) of S180 tumour-bearing mice. The findings shown in Figures 3 and 4 suggest that treatment with raw LGT decreases the amount of antioxidants and antioxidant enzymes, which results in cellular damage, while treatment with processed LGT reverses the high toxicity associated with LGT treatment to some degree.

### Processing with herbs reversed LGT-induced abnormal levels of TNF-α and IL-10 in liver and kidney

**Figure 5.**
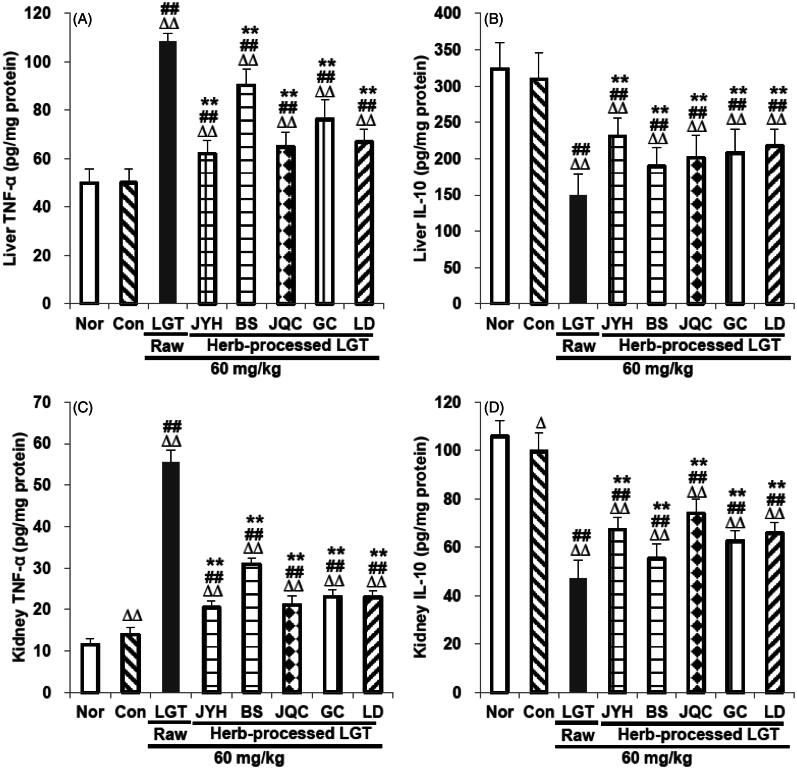
Effects of processed LGT treatment on TNF-α (A,C) and IL-10 (B,D) levels in the liver and kidney of LGT-exposed S180 tumour-bearing mice. Significant differences compared with the normal (Nor) group were designated as Δ*p* < 0.05 and ΔΔ*p* < 0.01, with the control (Con) group as ##*p* < 0.01, and with LGT raw product group as **p < 0.01.

We next examined the effects of processed LGT treatment on TNF-α (inflammatory cytokine) and IL-10 (anti-inflammatory cytokine) levels in the liver and kidney of LGT-exposed S180 tumour-bearing mice, in an attempt to link anti-inflammatory reactions to the apparent detoxification effect of treatment with processed LGT. In this study, inoculation of S180 tumour significantly increased kidney pro-inflammatory cytokine TNF-*α* (*p* < 0.01) (Figure 5(C)) and reduced kidney anti-inflammatory cytokine IL-10 (*p* < 0.05) (Figure 5(D)) without significant effects on liver TNF-*α* (Figure 5(A)) and IL-10 (Figure 5(B)) in mice. Administration of LGT raw product significantly increased TNF-*α* levels and reduced IL-10 levels (all *p* < 0.01) in liver and kidney of mice (Figure 5(A–D)), while treatments of its processed-herb products including JYHLGT, BSLGT, JQCLGT, GCLGT and LDLGT all significantly reversed the above excessively elevated TNF-*α* and decreased IL-10 levels (all *p* < 0.01) induced by LGT in S180 tumour-bearing mice (Figure 5). These results indicated that the anti-inflammatory reactions could involve the detoxification effects of LGT via processed with herbs including JYH, BS, JQC, GC and LD.

### Processing with herbs upregulated the expression of Nrf2 and HO-1

**Figure 6.**
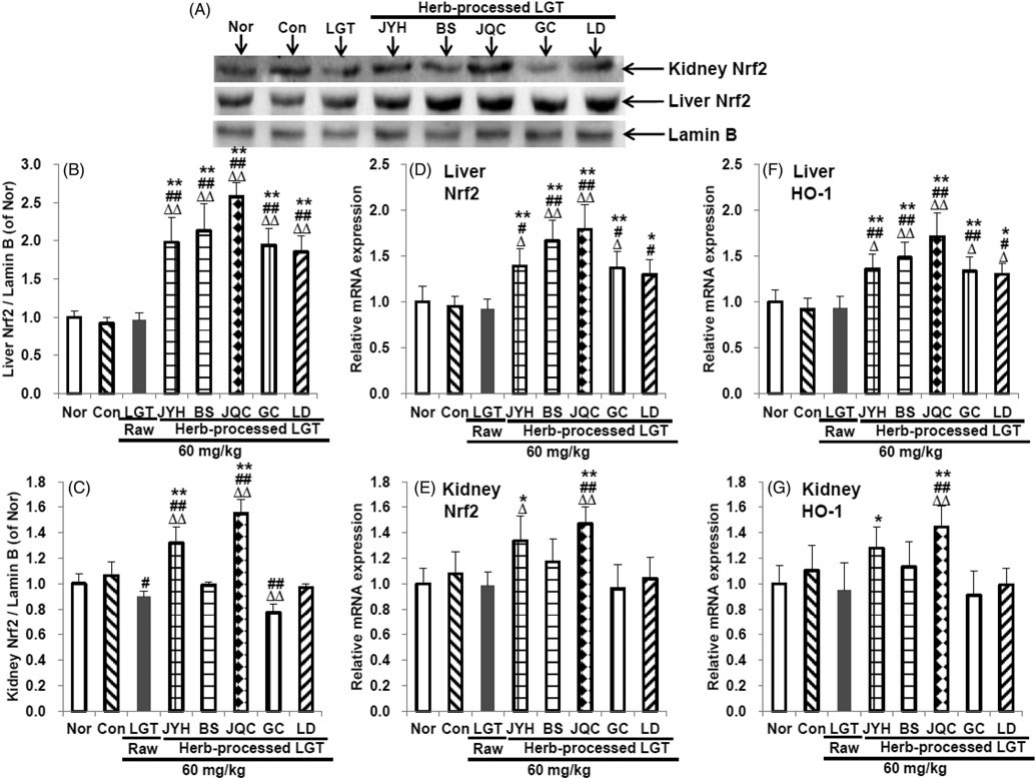
Effects of processed LGT treatment on protein expression of Nrf2 (A-C), and mRNA expression of Nrf2 (D,E) and HO-1 (F,G) in liver and kidney of LGT-exposed S180-bearing mice. Significant differences compared with the normal (Nor) group were designated as Δ*p* < 0.05 and ΔΔ*p* < 0.01, with the control (Con) group as #*p* < 0.05 and ##*p* < 0.01, and with LGT raw product group as *p < 0.05 and **p < 0.01.

In this study, inoculation of S180 tumour had no significant effects on the protein expression of Nrf2 (Figure 6(A–C)), and mRNA expression of Nrf2 (Figure 6(D,E)) and HO-1 (Figure 6(F,G)) in liver and kidney of mice. Administration of raw LGT significantly downregulated the protein expression of Nrf2 (Figure 6(A,C)) (*p* < 0.05) in kidney of S180-bearing mice without obvious effects on the protein expression of Nrf2 (Figure 6(A,B)) in liver, the mRNA expression of Nrf2 and HO-1 in liver and kidney (Figure 6(D–G)). Processing with herbs (JYH, BS, JQC, GC and LD), all significantly upregulated the protein expression of Nrf2 (Figure 6(A,B)) (all *p* < 0.01), and the mRNA expression of Nrf2 (Figure 6(D)) (*p* < 0.01, *p* < 0.01, *p* < 0.01, *p* < 0.01 and *p* < 0.05, respectively) and HO-1 (Figure 6(F)) (*p* < 0.01, *p* < 0.01, *p* < 0.01, *p* < 0.01 and *p* < 0.05, respectively) in liver of S180-bearing mice, compared with LGT raw product. In addition, processing with herbs (JQC and JYH) also significantly upregulated the protein expression of Nrf2 (Figure 6(A,C)) (*p* < 0.01 and *p* < 0.05, respectively), and the mRNA expression of Nrf2 (Figure 6(E)) (*p* < 0.01 and *p* < 0.05, respectively) and HO-1 (Figure 6(G)) (*p* < 0.01 and *p* < 0.05, respectively) in kidney of S180-bearing mice, compared with LGT raw product, while processing with herbs (BS, GC and LD) had no significant effects on them (Figure 6(A,C,E,G)). These results suggested that Nrf2 could probably participate in the detoxification mechanisms of LGT via processed with medicinal herbs including JYH, BS, JQC, GC and LD.

### Processing with herbs promoted LGT-produced antitumour activity

**Figure 7.**
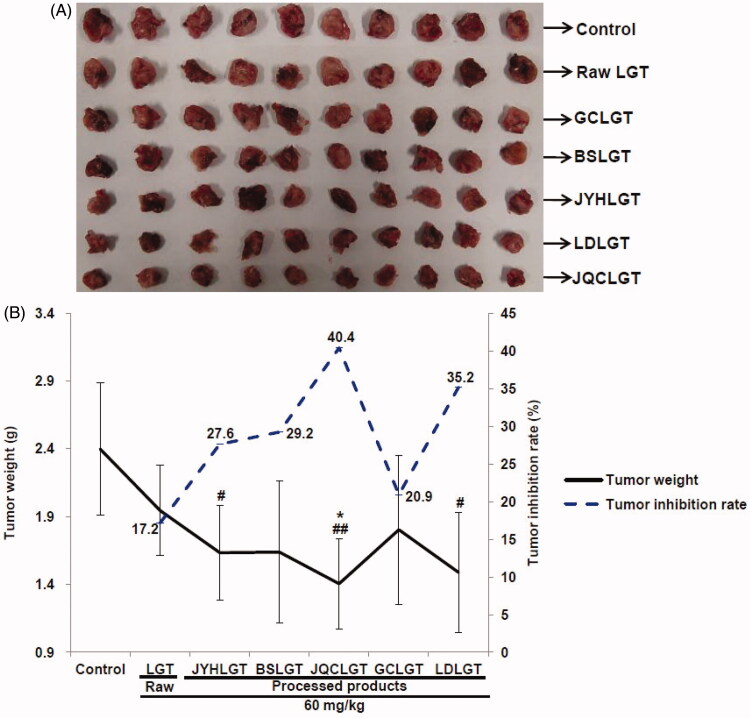
Effects of processed LGT treatment on the antitumour activity (A: Tumour picture and B: Tumour weight and inhibition rate) of LGT in S180 tumour-bearing mice. Significant differences compared with the control (Con) group as #*p* < 0.05 and ##*p* < 0.01, and with LGT raw product group as *p < 0.05.

We further observed the effect of processing with herbs on the antitumour activity of LGT. The results showed that administration of LGT raw product reduced the tumour weight with a 17.2% inhibition rate, while treatments of its processed-herb products including JYHLGT, BSLGT, JQCLGT, GCLGT and LDLGT all further reduced LGT-decreased tumour weight (Figure 7), with higher inhibition rates of 27.6, 29.2, 40.4, 20.9 and 35.2%, respectively. Among the above five processed products, JQCLGT significantly reduced LGT-decreased tumour weight (*p* < 0.05) (Figure 7) and increased the tumour inhibition rates by 23.2%.

## Discussion

Some potently toxic medicinal herbs, such as LGT (Liu et al. 2009; Bao and Dai 2011; Ge et al. 2013; Wang et al. 2016a), Strychni Semen (Maqianzi) (Guo et al. 2018), Arsenic trioxide (Pishuang) (Song et al. 2018) and so on, have excellent efficacy in the treatment of difficult diseases. How to reduce the toxicity of such toxic herbs with excellent curative effect without reducing their curative effects is one of the major issues of academic interest.

As for LGT, it has excellent curative effects including antitumours, but it is potently toxic and severely limits its clinical application. To this end, under the guidance of TCM theory, we conducted research on the detoxification mechanisms of LGT by processing with medicinal herbs with sweet properties (JYH, BS, JQC, GC and LD) and confirmed that processing with these herbs can all exhibit detoxification effects on LGT-induced hepatotoxicity and/or nephrotoxicity in S180 tumour-bearing mice and the mechanisms probably involved, at least in part, the Nrf2 antioxidant and anti-inflammatory processes.

A significant increase in serum ALT and (or) AST is indicative of liver injury (Li et al. 2015), and elevated serum Cr and (or) BUN are indicative of renal injury (Li et al. 2015). A recent study showed that LGT induced hepatotoxicity and nephrotoxicity in normal mice, evidenced by marked elevation in serum ALT, AST, BUN and Cr levels (Li et al. 2015). In this study, the administration of LGT raw product further significantly increased the levels of ALT, BUN and Cr in the serum of S180 tumour-bearing mice, while there was no statistically significant increase in the serum AST level. As to why there is no significant effect on the serum AST level in tumour-bearing mice after LGT administration, except that this may be the case, we speculate that the cause may be related to the animal’s strain, batch and individual differences, dose and time of administration, and so on. Nonetheless, our results suggested that the administration of LGT raw product significantly aggravated the hepatotoxicity and nephrotoxicity of S180 tumour-bearing mice to some extent. Fortunately, LGT-induced hepatotoxicity and nephrotoxicity in S180 tumour-bearing mice were reversed after treatments with its processed products prepared by JYH, BS, JQC, GC and LD, evidenced by the significant reduction of the above four serum biochemical markers and the improvement of liver and kidney pathological lesions.

In fact, studies have shown that JYH, BS, JQC, GC and LD and the main active extracts and compounds they contain have hepatoprotective and (or) kidney-protective effects (Sohn et al. 2003; Sun et al. 2008, 2010; Wang et al. 2012d; Liu et al. 2015; Yagmurca et al. 2015; Jung et al. 2016; Wang et al. 2016c; Xin et al. 2016; Xie et al. 2018; Ye et al. 2017). Therefore, the processed detoxification of the above five medicinal herbs on LGT-induced hepatotoxicity and nephrotoxicity is not only related to TCM theoretical support belong to them such as sweet and slow detoxification, antidote poisoning by the sweetness of the five flavours, mutual detoxification of seven emotions and so on, but also may be related to their bioactive properties of hepatoprotection and (or) renal protection. In addition, the TP content in the above five processed products decreased by 89.1, 81.9, 91.3, 65.7 and 85.0%, respectively, and the CEL content decreased by 84.8, 89.8, 96.3, 94.3 and 87.0%, respectively. The results suggested that the content of TP and CEL after processing was obviously reduced, which could be another important factor in reducing toxicity. In addition, both TP and CEL contents in JQC-processed product decreased the most than the other four processed products, which can probably explain to some extent why the JQC-processed product group had relatively lower levels of serum ALT, AST, Cr and BUN than the other four processed groups in this study. This may be further explained why JQC had a relatively better detoxification effect on hepatotoxicity and nephrotoxicity caused by LGT in this study.

Considering that the toxicity caused by LGT was related to abnormal antioxidant capacity and inflammatory response (Li et al. 2015; Wang et al. 2015), and at the same time these medicinal herbs (JYH, BS, JQC, GC and LD) had been reported to have antioxidant and anti-inflammatory properties (Gu et al. 1988; Lee et al. 2005; He and Dai 2011; Wu et al. 2011; Chen et al. 2012; Guo et al. 2014; Luo et al. 2016; Tóth et al. 2016), so next we tried to explore the potential mechanism of detoxification by analyzing some antioxidant and inflammation related indicators. In this study, inoculation of S180 tumour significantly reduced the levels of antioxidants GSH and GST in the liver and kidney, and at the same time caused excessive levels of inflammatory mediator TNF-α and too low levels of anti-inflammatory mediator IL-10. To a large extent, it was suggested that inoculation of the tumour caused oxidative damage and/or inflammatory damage of the liver and kidney. LGT raw product administration significantly reduced the levels of antioxidants GSH, GST, GPx, SOD and CAT in the liver and kidney of S180 tumour-inoculated mice and meanwhile significantly increased the inflammatory mediator TNF-α level as well as decreased the anti-inflammatory mediator IL-10 level. These results suggested that LGT caused or even aggravated oxidative damage and inflammatory damage in the liver and kidney of tumour mice. Fortunately, after taking LGT-processed products (JYHLGT, BSLGT, JQCLGT, GCLGT and LDLGT), compared with the LGT raw product group, the indicators of the above abnormalities caused by LGT were all significantly reversed in the liver and kidney of tumour mice, suggesting that the antioxidant and anti-inflammatory processes could be probably involved in the detoxification mechanism of LGT via processed with medicinal herbs including JYH, BS, JQC, GC and LD. Actually, it was reported that these five herbs had good antioxidant and anti-inflammatory properties (Gu et al. 1988; Lee et al. 2005; He and Dai 2011; Wu et al. 2011; Chen et al. 2012; Guo et al. 2014; Luo et al. 2016; Tóth et al. 2016). Therefore, to some extent, the detoxification effects of these five herbs on LGT via processing could be partially attributed to their own antioxidant and anti-inflammatory properties. Besides, the effects of these five processed products on these antioxidant and inflammation-related indicators are generally the same, but to varying degrees.

Moreover, considering Nrf2, an antioxidant key transcription factor, plays an important role in both antioxidant and anti-inflammatory processes (Wang et al. 2018b), we further observed the detoxification mechanism of LGT by analyses of Nrf2 protein and mRNA expression in liver and kidney of tumour mice. In this study, processing with medicinal herbs (JYH, BS, JQC, GC and LD) promoted Nrf2 nuclear accumulation in LGT-exposed tumour mice, evidenced by significantly upregulated Nrf2 protein levels, and mRNA levels of *Nrf2* and its downstream target gene *HO-1*, suggesting that Nrf2 activation in liver could probably participate in the above medicinal processed-herb detoxification mechanism of LGT. As for expression analysis in the kidney, only JQC and JYH concocted LGT promoted Nrf2 nuclear aggregation in tumour mice, evidenced by significantly upregulated Nrf2 protein levels, and Nrf2 and HO-1 mRNA levels, suggesting that Nrf2 activation in kidney could probably participate in the JQC and JYH concocted detoxification mechanism of LGT. However, in this study, BS, GC and LD concocted LGT did not cause activation of Nrf2 in kidney, suggesting that the detoxification of LGT by processing with medicinal herbs (BS, GC, and LD) may not be regulated by Nrf2 in kidney. In addition, the lack of detection of cytosolic Nrf2 protein expression, as well as the lack of the measurement of protein expression on other related signalling molecules such as HO-1 in the Nrf2 signalling pathway, is one of the limitations of this study.

After confirming that the processing with medicinal herbs can have detoxifying effect on LGT, we further examined the effect of concocting on the antitumour activity of LGT. In this study, administration of LGT raw product reduced the tumour weight with a 17.2% inhibition rate, while processing with medicinal herbs (JYH, BS, JQC, GC and LD) all further decreased LGT-decreased tumour weight, concomitant with the rates of tumour inhibition increased by 10.4, 12, 23.2, 3.7 and 18%, respectively. That is to say, not only the detoxification effect on LGT was obtained by processing with medicinal herbs (JYH, BS, JQC, GC and LD), but also the antitumour activity of LGT is also increased, especially the synergistic effect of JQC concocted LGT is the strongest, followed by LD, BS, JYH and GC. Actually, as reported, JQC-contained quercetin and rutin, LD-contained peptides, JYH-contained luteolin and chlorogenic acid, GC-contained glycyrrhizin and isoangustone A, and BS-contained paeoniflorin all exerted antitumour properties (Thirugnanam et al. 2008; Seon et al. 2012; Alonso-Castro et al. 2013; Chen et al. 2017; Yan et al. 2017; Li et al. 2018a, 2018b; Ouyang et al. 2018). Therefore, we speculate that the synergistic effect of LGT by processing with medicinal herbs (JQC, LD, JYH, GC and BS) may be attributed in part to the interaction of the antitumour active ingredients contained in these five herbs with the active ingredients in LGT. In addition, Nrf2-based antioxidant and anti-inflammatory defence processes may also contribute to their synergistic effects. However, the diameter of the tumour was not detected, so that the evaluation of antitumour efficacy was not comprehensive enough. Therefore, this is another limitation of our research. In addition, we did not detect tumour markers to explore antitumour mechanisms, which may be the third limitation of our study.

Together, thorough processing with medicinal herbs (JQC, LD, JYH, GC and BS) exhibited detoxification effect on the hepatotoxicity and nephrotoxicity caused by LGT, and the mechanisms could be at least partly attributed to upregulation of Nrf2 and its downstream HO-1 signal, thereby enhancing antioxidant defences, and inhibiting inflammation. In addition, the antitumour activity of each processed product of LGT increased to different degrees compared with its raw product, among which JQC processed product have the strongest synergistic effect. According to the results we have obtained, we can provide some tips or suggestions for clinical use of LGT. First, clinically, we can consider the use of LGT-processed products (JYHGT, BSLGT, JQCLGT, GCLGT and LDLGT) to reduce its toxicity, while at the same time its activity cannot be reduced or even can be enhanced, although this requires more evidence to support. In addition, we can also consider the use of these medicinal processed-herb products to reduce the intake of LGT, thereby reducing the risk of poisoning, but its curative effect is not attenuated, although this consideration requires more evidence to support. Finally, due to the strong toxicity of LGT, although the toxicity of LGT-processed products of the present study was reduced, there are still safety risks of medication.
